# Structural Characteristics and Assembly Mechanisms of Soil Microbial Communities under Water–Salt Gradients in Arid Regions

**DOI:** 10.3390/microorganisms11041060

**Published:** 2023-04-18

**Authors:** Guang Yang, Lamei Jiang, Wenjing Li, Eryang Li, Guanghui Lv

**Affiliations:** 1 College of the Ecology and Environment, Xinjiang University, Urumqi 830017, China; 107552000826@stu.xju.edu.cn (G.Y.); jianglam0108@126.com (L.J.); liwenjing0624@163.com (W.L.); eryang_l@stu.xju.edu.cn (E.L.); 2 Key Laboratory of Oasis Ecology of Education Ministry, Xinjiang University, Urumqi 830046, China

**Keywords:** arid zone, microbial diversity, co-occurrence network, community assembly

## Abstract

Exploring the structural characteristics of arid soil microbial communities and their assembly mechanisms is important for understanding the ecological characteristics of arid zone soils and promoting ecological restoration. In this study, we used Illumina high-throughput sequencing technology to study soils in the arid zone of the Lake Ebinur basin, determined the differences among soil microbial community structures in the study area under different water–salt gradients, and investigated the effects of environmental factors on microbial community structure and assembly mechanisms. The results show the following: the microbial community alpha diversity exhibited a significantly higher low water–salt gradient (L) than high water–salt gradient (H) and medium water–salt gradient (M). The pH was most strongly correlated with soil microbial community structure, where the alpha diversity indices of the bacterial community and fungal community were significantly negatively correlated with pH, and the Bray–Curtis distance of bacterial community was significantly positively correlated with pH (*p* < 0.05). The complexity of bacterial community co-occurrence networks showed a significantly higher L than H and M, and the complexity of fungal community co-occurrence network showed a significantly lower L than H and M. The cooperative relationship of H and M in the co-occurrence networks was stronger than that of the L, and the key species of the microbial co-occurrence network were different under different water–salt gradients. Stochastic processes dominated the assembly mechanism of the microbial community structure of soil, and the explanation rates of deterministic and stochastic processes were different under different water–salt gradients, with the highest explanation rate of stochastic processes on the L accounting for more than 90%. In summary, the soil microbial community structure and assembly mechanisms significantly differed across water–salt gradients, and these findings can help provide a reference for further research on soil microbiology in arid zones.

## 1. Introduction

Desertified lands are widely distributed on Earth, with sparse vegetation and perennial water scarcity in desert areas, which account for about 40% of the land area [1]. Global changes have caused an increase in the aridity of many desert ecosystems, which further exacerbates the process of soil erosion and desertification in arid zones and has led to a severe degradation of 10-20% in global desert ecosystems [2,3]. Despite the extreme conditions of desert ecosystems, they are endowed with a large number of biological resources [4]. Many field studies around the world have shown that increased aridity can have a significant effect on the above- and below-ground biotic community properties as well as on abiotic factors of desert zone ecosystems [5,6,7]. Soil microbial communities are highly diverse and can rapidly respond to environmental changes [8]. The results of a study by Leng et al. [9] on the relationship between inter- and intra-root microbial communities and various soil environmental factors showed that multiple environmental factors have significant effects on microbial community composition and diversity. Li et al. [10] analyzed the relative evolutionary rates of microbial communities from six different natural environments and showed that the evolutionary patterns of microbial communities are mainly influenced by environmental conditions. Soil microorganisms can regulate the functions and properties of ecosystems and are of great significance for the stability and sustainability of ecosystems [11,12].

With the iterative update of high-throughput sequencing technology, a large number of fruitful research findings have been published, improving our understanding of the formation mechanisms of soil biome structure and diversity [13]. The study of microorganisms has gradually progressed from community distribution patterns to community assembly mechanisms within communities. The community assembly mechanism determines the process of microbial community formation and the direction of succession [14]. Vellend [15] proposed a process-based theoretical framework for community ecology in 2010, suggesting that selection, drift, dispersal, and diversification determine microbial community assembly, and that combinations of four processes to varying degrees can be used to explain all community assembly mechanisms. On this basis, Stenge et al. [16] combined the approaches of a phylogenetic zero model and taxonomic unit zero model to determine the ratios of four ecological processes—homogeneous selection, heterogeneous selection (deterministic), homogeneous diffusion, and diffusion limitation (stochastic)—and quantitatively resolve the microbial community assembly mechanisms. In recent years, many scholars have successfully conducted studies on microbial community assembly using this approach [17,18,19,20]. For desert areas, it has been shown that the soil microenvironment changes with the water–salt gradients, further altering the structural composition of soil microbial communities [21]. However, there is a paucity of research on the patterns of soil microbial community assembly in desert areas under water–salt gradients, which limits our understanding of microbial community composition and diversity formation mechanisms in desert ecosystems. Therefore, it is important to explore the ecological processes of soil microbial community assembly in desert areas, which can provide some ideas to solve the environmental problems of desertification.

The Lake Ebinur basin is located in the most depressed and largest water–salt convergence center in the western part of the Junggar Basin in China and is part of a typical temperate continental arid climate. The climate is extremely dry, and precipitation is scarce [22]. The soils in the reserve are mostly sandy and clayey [23], and soil salinization is more serious. Some studies have shown that the water–salt content of soils in the riparian zone of the Achiksu River, which is located in the reserve, is high and decreases with increasing distance from the river [24]. Thus far, scholars have been conducting more research on the structure of soil microbial communities and their relationship with the environment in the arid zone of the Lake Ebinur basin, but most of the resulting studies mainly focus on the response of microbial communities to a single environment, such as land type, soil physicochemical factors, seasonal changes, etc., rarely addressing the interaction relationship within the soil microbial communities and the joint response mechanism of environmental factors [21,25,26,27,28,29]. Based on this, the present study was conducted to explore the process of soil microbial community assembly and influencing factors under different water–salt gradients via a field investigation and soil sample analysis in a typical desert ecosystem in Lake Ebinur Wetland Nature Reserve in order to answer the following scientific questions: (1) What are the differences in the community composition of the soil microbiome in an arid zone under different water–salt gradients? (2) What is the pattern of interaction between soil microbial groups in different water–salt gradients? (3) Which process dominates in the soil microbial community assembly process under different water–salt gradients? The following hypotheses were addressed: (1) The composition of soil microbial communities and their internal interactions in the study area vary considerably in terms of water and salt gradients due to the different physiological structural characteristics and water dependence of various soil microorganisms [30,31,32] and the vulnerability of microbial communities to salt stress in saline soils [33,34]. (2) Moisture and salinity are important environmental constraints on soil microbial community structure and interactions, and therefore the relative importance of stochastic and deterministic processes in soil microbial community construction will differ markedly across water and salt gradients [34,35,36].

## 2. Materials and methods

### 2.1. Overview of the Study Area

The Ebinur Lake Wetland National Nature Reserve (82°36′–83°50′ E, 44°30′–45°09′ N) is located in the northwest of the Xinjiang Uygur Autonomous Region, southwest of the Junggar Basin, with a total area of 2670.85 square kilometers. Due to its harsh climatic conditions and unique topographic conditions, the Ebinur Lake basin possesses a complex and diverse regional landscape of rocky desert, gravel desert, desert, salt desert, marsh and salt lake. The annual evaporation is over 1600 mm, and the annual precipitation is about 100 mm. Soil salinity is high and alkalinity is strong, and the average conductivity of shallow soil (0–10 cm) is 5.41 mS/cm, the average pH value is 8.77, and the average water content is 7.19% [37]. Since surface vegetation is sparse and unevenly distributed, surface, subsurface, and water recharge mainly originates from mountainous areas, and the phenomenon of soil erosion is more common.

### 2.2. Method

#### 2.2.1. Sample Setting

**Figure 1:**
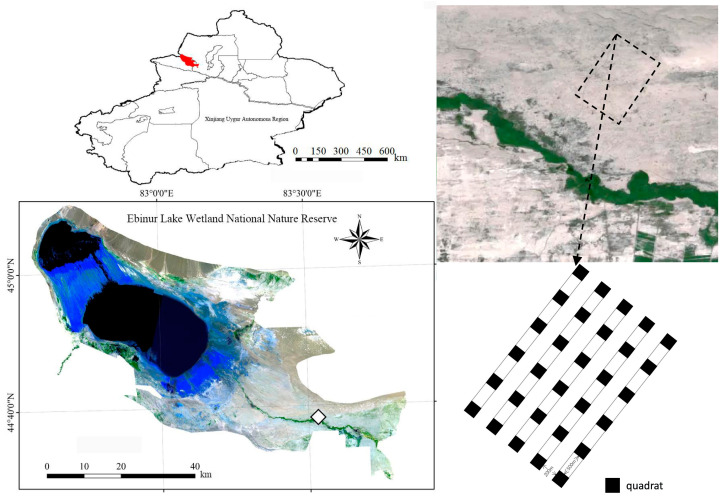
The schematic diagram of sampling and sample.

In the desert area of Ebinur Lake National Nature Reserve, five 20 m × 2620 m sample strips were set up perpendicular to the Achiksu River, with a 200 m interval between adjacent sample strips, and six 20 m × 20 m sample squares were laid in each sample strip, with a 500 m interval between each sample square, totaling 30 sample squares (Figure 1), which were named C1–C30, in order. The geographical coordinates and elevation of each sample plot were recorded via the GPS (center point) elevation of each sample site. Starting with sample square C1, each sample square was sampled in turn.

#### 2.2.2. Soil Sample Collection

In the 20 m × 20 m sample plots, one well-grown plant of each species was selected to collect 0–10 cm of sub-canopy soil (collection box 60 mL), which was placed into 1 L sterile sampling bags and mixed. One sample was placed into three 50 mL centrifuge tubes for retaining soil samples, and the other samples were placed into three 5 mL lyophilized tubes for sending samples for determination. Before sampling, masks were worn, profiles were dug according to species, visible impurities were removed with a shovel, and then sterile gloves were worn to collect samples using 60 mL sampling boxes. The lyophilized tubes were placed into liquid nitrogen tanks for temporary storage for the subsequent determination of microbial composition.

#### 2.2.3. Determination of Physical and Chemical Parameters

Three sampling points were randomly selected within a 20 m × 20 m sample square for sampling. First, the soil was collected with an empty aluminum box weighed and marked with the sample square number. The fresh soil was weighed with a one-ten-thousandth accuracy balance, and then brought back to the laboratory for drying and weighing to obtain the dry soil weight, which was used to calculate the soil water content. The soil was collected from 0 to 10 cm under the canopy of the plant consistent with microbial sampling and placed in a self-sealing bag (about 200 g) for the determination of soil physical and chemical properties, and the indexes and methods were determined, as shown in Table S1.

#### 2.2.4. Soil Microbial Assay

DNA was extracted from 30 sets of samples collected using FastDNA Spin Kit (MP Biomedicals, Santa Ana, CA, USA) The total DNA of the extracted soil microorganisms was amplified by PCR and a sequencing library was created (polymerase, 20 μL reaction system). The primers for bacterial 16S rDNA V3-V4 amplification were 338F and 806R. Fungal ITS was amplified using primers 1737-F and 2043-R for the ITS1 region of TS1 rRNA gene. After the pre-experiment results were qualified, the Illumina MiSeq PE250 platform was used for sequencing analysis (Shanghai Majorbio Biomedical Technology Co., Shanghai, China).

The raw sequencing data were spliced, filtered, clustered, and annotated with species. QIIME 2 software, Mothur, was used to calculate the alpha diversity of microbial communities in the samples, such as the actual number of OTU observations (OTUs), Chao index and Shannon index were used.

#### 2.2.5. Statistical Analysis

Soil water content and soil salinity were analyzed using the “Claster” package in R 4.2.1 for 29 samples (soil data from sample C17 were missing) by K-means cluster analysis. One-way ANOVA was performed to compare the differences in soil physicochemical properties and microbial composition under different water–salt gradients. Pearson correlation analysis was performed to correlate soil physicochemical properties and microbial diversity, and principal coordinate analysis (PCoA) was used to explore the differences in soil bacterial and fungal communities under different water–salt gradients. Bray–Curtis distance and βMNTD distance matrices of species were Mantel-correlated with each soil chemical property, respectively, and the main environmental factors affecting the soil microbial community structure were analyzed using RDA. One-way ANOVA and correlation analyses were performed in SPSS 26.0, while PCoA, Mantel test and RDA analyses were performed in the “vegan” package in R 4.2.1.

To improve the reliability of the co-occurrence network, firstly, the co-occurrence network of bacteria and fungi on different water–salt gradients was constructed at the genus level by screening the samples at a discovery rate of no less than 10%, and using r > 0.7 and *p* < 0.05 as the thresholds for Spearman correlations between species. The topological characteristics of the co-occurrence networks were characterized by the following parameters: nodes, edges, degrees—the number of edges connected to each node; clustering coefficient—indicates the degree to which nodes tend to cluster together; and modularity. When the modularity index is higher, it means the network has more modularity (the higher the modularity index, the lower the complexity of the network). Betweenness centrality (the ability of a node to control the shortest path to other nodes in the network, which can be used to identify key species in the network) [38,39] was calculated using the “igraph” package in R software. Finally, Gephi (v 0.9.2) was used to visualize the microbial co-occurrence network under different water–salt gradients, with node sizes set by abundance and nodes colored by gate classification level, and edge colors set by source node.

A null model was used to analyze the mechanisms of soil microbial community assembly under different water–salt gradients [16]. Firstly, the between-community mean nearest taxon distance (βMNTD), β-nearest taxon index (β-NTI), and Raup–Crick matrix (RCbray) of soil microbial communities under different water–salt gradients were calculated using the Majorbio Cloud Platform (www.majorbio.com). β-NTI > 2 indicates that the community assembly process is heterogeneous selection in the deterministic process, and β-NTI < −2 indicates that the community assembly process is homogeneous selection in the deterministic process. When |β-NTI| < 2 and RCbray > 0.95, it indicates that the community assembly process is dispersal limitation in the stochastic process. When |β-NTI| < 2 and RCbray < −0.95, it indicates that the community assembly process is homogenizing dispersal in the stochastic process. When |β-NTI| < 2 and |RCbray| < 0.95, it indicates that the community assembly process is undominated processes in the stochastic process. The undominated processes include weak selection, weak diffusion, diversification and drift processes.

## 3. Results and Analysis

### 3.1. Water–Salt Gradients and Physicochemical Properties of Soils

**Figure 2:**
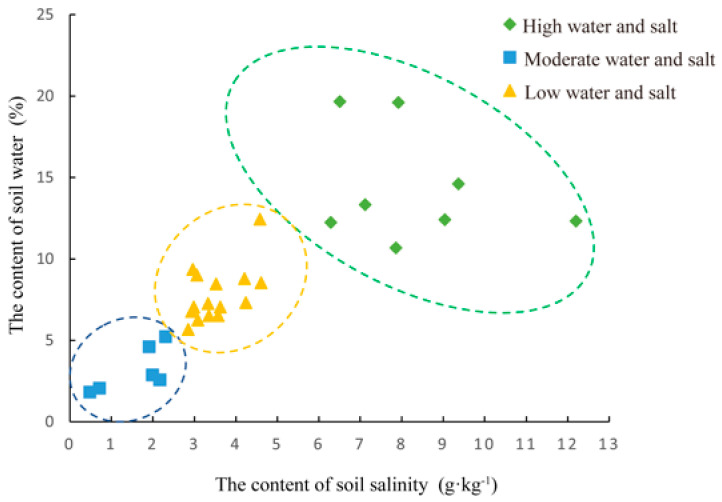
The Clustering results of water-salt gradient in sample plots.

The final results of the clustering of soil water–salt gradients in the samples were the following: eight samples for the high water and salt (H) gradient, 15 samples for the medium water and salt (M) gradient, and six samples for the low water and salt (L) gradient (Figure 2, Table S2). One-way ANOVA of soil water content and salinity of the clustered samples (Table S3) showed that the mean values of soil water content varied between 3.193% and 14.353% and soil salinity varied between 1.596 g·kg^−1^ and 8.289 g·kg^−1^ for the three gradients. There were significant differences in soil water content and soil salinity between the three gradients (*p* < 0.05).

The characteristics of soil physicochemical properties at different water–salt gradients are shown in Table S4. The soil had a weak alkalinity at all water–salt gradients, and the alkalinity increased with increasing water–salt content, and the pH values were significantly different between the gradients. This indicates that soil nutrients increased with increasing water and salt content. Based on the variation of soil enzyme activities between water–salt gradients (Table S5), it can be seen that, except for alkaline phosphatase (AKP), leucine aminopeptidase (LAP), β-glucosidase (β-GC), and N-acetyl-β-D-glucosidase (NAG) enzyme activities showed a tendency to decrease with decreasing water and salt content, and the enzyme activities in H were significantly higher (*p* < 0.05) than those in the M and L.

### 3.2. Soil Microbial Community Composition under Different Water–Salt Gradients

**Figure 3:**
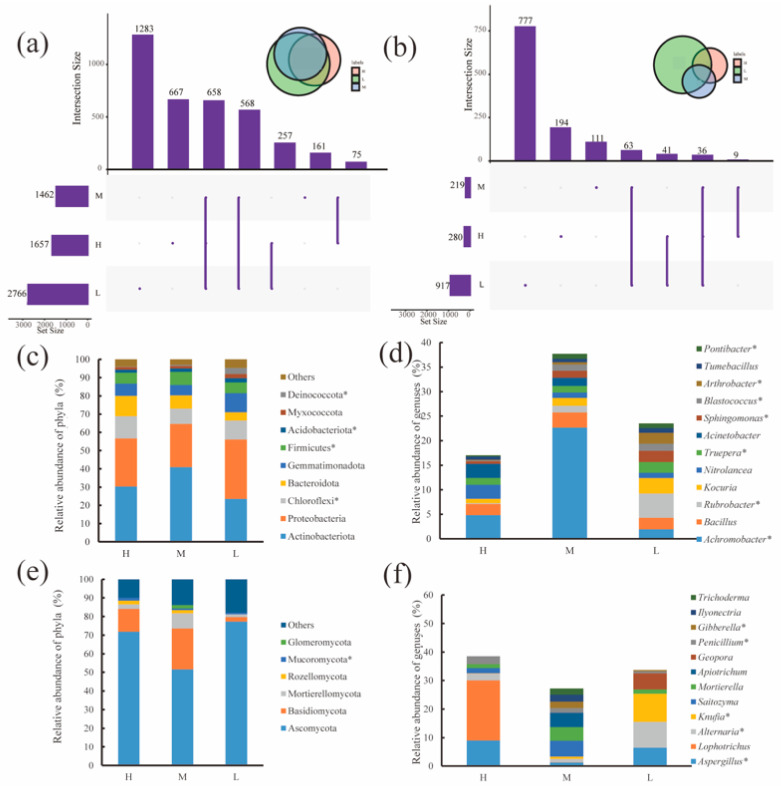
Distribution of microbial communities at the OTU, phylum and genus level under different water-salt gradients. Note: H, M and L denote high, medium and low water-salt gradients, respectively; (**a**,**c**,**d**) indicate the distribution of bacterial communities at the OTU, phylum, and genus levels, (**b**,**e**,**f**) indicate the distribution of the fungal communities at the OTU, phylum and genus levels. * indicates significant differences in microorganisms between groups (*p* < 0.05).

Bacteria and fungi had 658 and 36 OTUs stably present in the water–salt gradients, respectively, with 667, 161 and 1283 OTUs unique to bacteria and 194, 111 and 777 OTUs unique to fungi in the H, M and L(Figure 3a,b). For the soil bacterial community, Proteobacteria (synonym Pseudomonadota) and Actinobacteriota (synonym Actinobacterium) had the highest relative abundances of 23.48–40.95% and 23.65–32.63%, respectively. Actinobacteriota had the highest relative abundance on the H and M, and Proteobacteria had the highest relative abundance on the L. In addition, the relative abundances of Chloroflexi, Firmicutes(synonym Finnicutes), Acidobacteria, Myxococcota and Deinococcota significantly differed (*p* < 0.05) in the water–salt gradients.

The variation in the relative abundance at the level of soil fungal phylum in different water–salt gradients is shown in Figure 3c. Ascomycota and Basidiomycota were the predominant phyla, with both accounting for more than 70% of the relative abundance. The relative abundance of Ascomycota was 51.58–77.30% and was significantly lower on the M than on the L. The relative abundance of Mucoromycota showed a significant decrease (*p* < 0.05) with decreasing water–salt gradient. In addition, Mortierellomycota, Rozellomycota, and Glomeromycota are also major fungal taxa, with Rozellomycota found only in the M and L and Glomeromycota found only in H and M.

The changes in the genus level of soil bacteria at different water–salt gradients are shown in Figure 3e, where the relative abundance of Achromobacter was significantly higher in the M than in the H and L (*p* < 0.01); the relative abundance of Rubrobacter(synonym Erythrobacter), Truepera, Sphingomonas, Blastococcus, Arthrobacter and Pontibacter showed a significant increasing trend (*p* < 0.05) with a decreasing water–salt gradient. Kocuria showed a trend of increasing relative abundance with the decreasing water–salt gradient. Nitrolancea and Acinetobacter showed a trend of decreasing relative abundance with decreasing water–salt gradient.

The variation of soil fungal genera at the level of different water–salt gradients is shown in Figure 3f. The distribution of these genera on the water–salt gradient varied somewhat, among which Aspergillus, Alternaria, Knufia, Penicillium, and Gibberella varied significantly with the water–salt gradients. In addition, Apiotrichum, Ilyonectria and Trichoderma were found only in the M.

### 3.3. Diversity Characteristics of Soil Microbial Communities under Different Water–Salt Gradients

**Figure 4:**
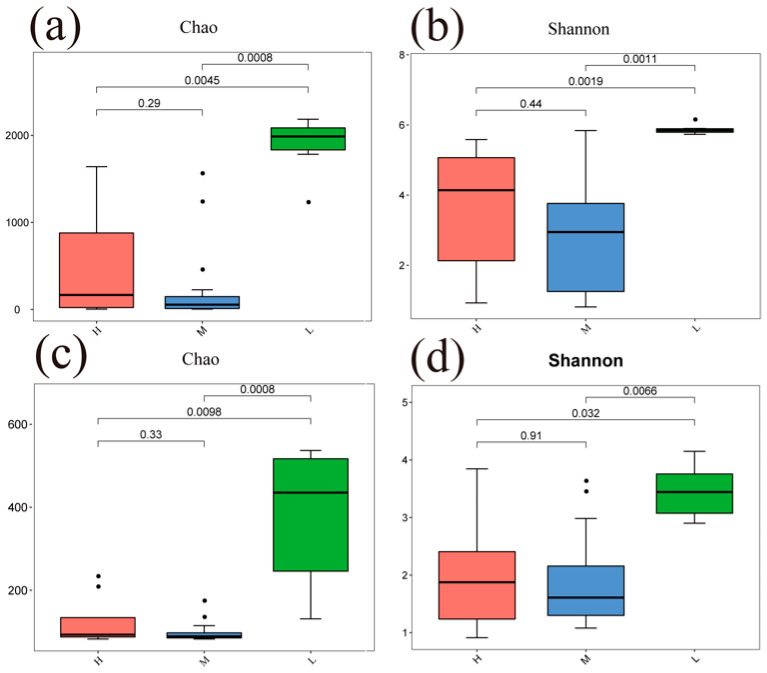
Alpha diversity of soil microorganisms under different water-salt gradients. Note: H, M and L denote high, medium and low water-salt gradients, respectively; (**a**,**b**) indicate the distribution of bacterial communities α-diversity among different water-salt gradients; (**c**,**d**) indicate the distribution of fungal communities α-diversity among different water-salt gradients.

**Figure 5:**
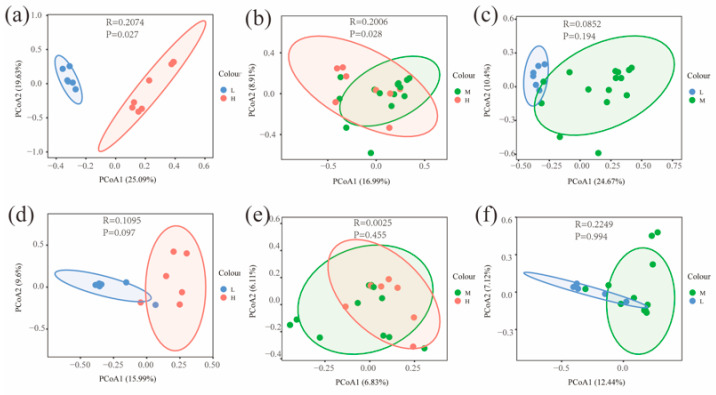
Beta diversity of soil microorganisms at different water-salt gradients. Note: H, M and L denote high, medium and low water-salt gradients, respectively; (**a**–**c**) represent PCoA analysis between different water-salt gradients of the bacterial communities; (**d**–**f**) represent PCoA analysis between different water-salt gradients of the fungal communities.

The Chao and Shannon indices of bacterial and fungal communities in the L were significantly different from both the H and M (*p* < 0.05) (Figure 4). A principal coordinate analysis (PCoA) based on Bray–Curtis distance for soil bacterial and fungal communities at different water–salt gradients, in which bacterial communities were completely separated at H and L, with the first principal axis PCoA1 and the second principal axis PCoA2 explained a total of 44.72% of the total variation in both and significant differences between H and L (*p* < 0.05) (Figure 5). The L and M of the bacterial community were statistically different (*p* < 0.05), although they were crossed (Figure 5).

### 3.4. Main Environmental Factors Affecting the Structure of Soil Microbial Communities

Bacterial OTUs were most strongly correlated with soil environmental factors, which included significant positive correlations with SOC, LAP, β-GC, NAG, and AKP (*p* < 0.05) (Table 1); pH was most strongly correlated with soil bacterial community structure, with the Chao index showing a highly significant negative correlation with pH (*p* < 0.01), the Shannon index showing a significant negative correlation with pH (*p* < 0.05) and the Bray–Curtis distance showing a significant positive correlation with pH (*p* < 0.05) (Table 1).

The correlations between fungal community structure and soil environmental factors were analyzed (Table 2). The results show that the βMNTD distance was the most strongly correlated with soil environmental factors, with significant positive correlations with SAP, SOC, LAP, β-GC, NAG and AKP, and highly significant positive correlations with SAN (*p* < 0.01); OTUs, Chao, and Shannon indices were highly significantly negatively correlated with pH (*p* < 0.01).

**Figure 6:**
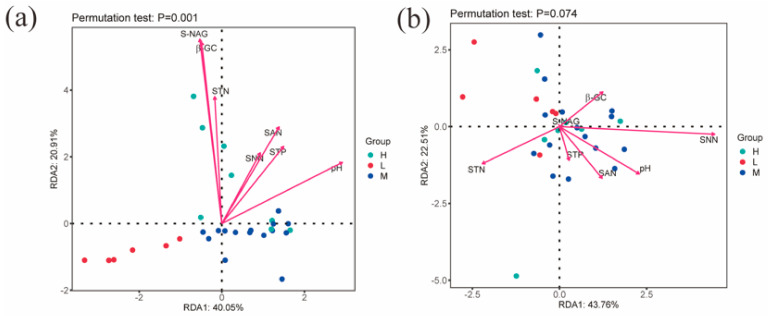
Redundancy analysis of soil factors and microbial community composition. Note: (**a**) indicates the RDA analysis of environmental factors and soil bacterial communities; (**b**) indicates the RDA analysis of environmental factors and soil fungal communities; S-NAG indicates N-acetyl-β-D-glucosidase, β-GC indicates β-glucosidase, STN indicates total nitrogen, SNN indicates nitrate nitrogen, SAN indicates ammonium nitrogen, and STP indicates total phosphorus; H, M and L denote high, medium and low water-salt gradients, respectively.

The RDA results show that the first and second major axes together explained 60.96% of the changes in bacterial community structure, and based on the length of the projection of environmental factors on the axes as well as the angles, it can be seen that pH, NAG, and GC are important factors affecting the soil bacterial community (Figure 6a). The first and second major axes together explained 66.27% of the changes in fungal community structure, and pH and STN are important factors affecting the soil fungal community (Figure 6b).

### 3.5. Co-Occurrence Network Characteristics of Microbial Communities under Different Water–Salt Gradients

**Figure 7:**
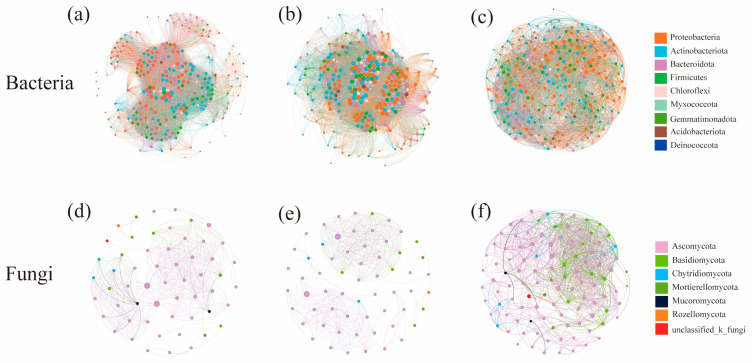
Co-occurrence network of soil microorganisms under different water-salt gradients. Note: (**a**–**c**) denote the co-occurrence networks of bacterial communities; (**d**–**f**) denote the co-occurrence networks of fungal communities; nodes indicate species at the genus level, node size indicates the magnitude of the degree, and node color indicates a different phylum; a line between nodes indicates that two nodes are correlated.

The co-occurrence networks of bacteria under different water–salt gradients were constructed at the genus level. The complexity of the bacterial co-occurrence network was significantly higher on the H and M than on the L (Figure 7a–c, Table 3). The negative correlations between nodes were significantly higher on the L than on the H and M. When analyzing the betweenness centralities of bacterial community co-occurrence network nodes, the core nodes differed under different water–salt gradients, and the three key species with the highest connectivity were Woeseia, Haliangium, and norank_f__Balneolaceae for the H; Paracoccus, norank_f __Microtrichaceae, and Microvirga for the M; and unclassified_c__Bacteroidia, Methyloceanibacter, and Gemmatimonas for the L.

The co-occurrence networks of fungi under different water–salt gradients were constructed at the genus level, and it could be observed that the complexity of the co-occurrence network was significantly higher on the L than on the H (Figure 7d–f, Table 3). The analysis of the characteristics of each network node revealed different core nodes on different gradients, and the three key species with the highest connectivity were Chaetomium, Neocamarosporium and Lophotrichus for the H; Engyodontium, Alternaria and unclassified_o_pleosporales for the M; and Alternaria, Sigarispora and Chaetosphaeronema for the L. Except for Chaetomium, Engyodontium, Sigarispora and Chaetosphaeronema, the other key species were all genera in the top 30 in terms of abundance.

**Figure 8:**
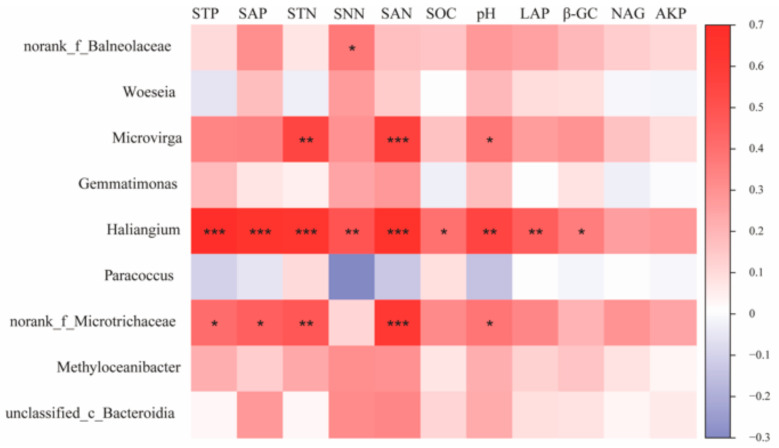
Correlation of bacterial keystone species in co-occurrence networks with environmental factors. Note: *, **, and *** in the figure indicate *p* < 0.05, *p* < 0.01, and *p* < 0.001, respectively; The values on the axis indicate R-value.

Correlation analysis of key species of bacterial communities under different water–salt gradients with environmental factors was performed (Figure 8). The results show that Haliangium, the key species of bacterial communities under H, showed significant positive correlations with STP, SAP, STN, SNN, SAN, SOC, pH, LAP, and β-GC (*p* < 0.05), and norank_f__Balneolaceae showed significant correlations with SNN (*p* < 0.05). The bacterial community key species, norank_f__Microtrichaceae, under the L, showed significant positive correlations (*p* < 0.05) with environmental factors such as STP, SAP, STN, SAN, SOC, and pH. The key species, Microvirga, under the L showed significant positive correlations (*p* < 0.05) with STN, SAN, and pH.

**Figure 9:**
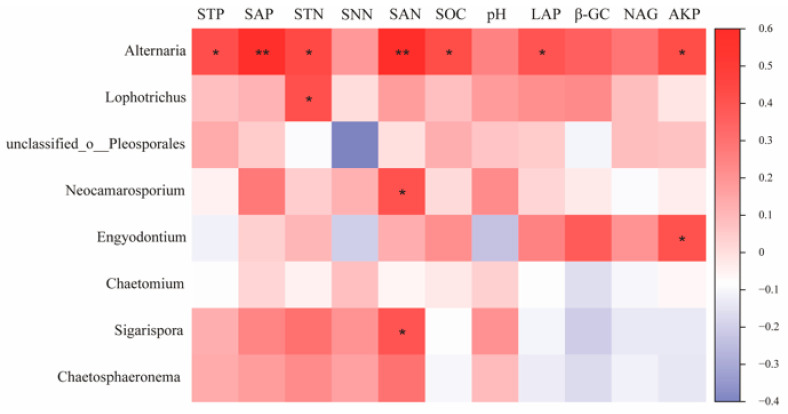
Correlation of fungal keystone species with environmental factors in co-occurrence networks. Note: * and ** in the figure indicate *p* < 0.05 and *p* < 0.01.

The results of the correlation analysis between key species of the fungal community and environmental factors (Figure 9) showed that Neocamarosporium, a key species on the H, showed a significant positive correlation with SAN (*p* < 0.05) and Lophotrichus with STN (*p* < 0.05). Engyodontium, a key species on the M, was significantly positively correlated with AKP. Alternaria, as a common keys species on the M and L, was significantly positively correlated with STP, SAP, STN, SAN, SOC, LAP, and AKP (*p* < 0.05). Sigarispora, as a keys species on the L, was significantly positively correlated with SAN (*p* < 0.05).

### 3.6. Soil Microbial Community Assembly Process

**Figure 10:**
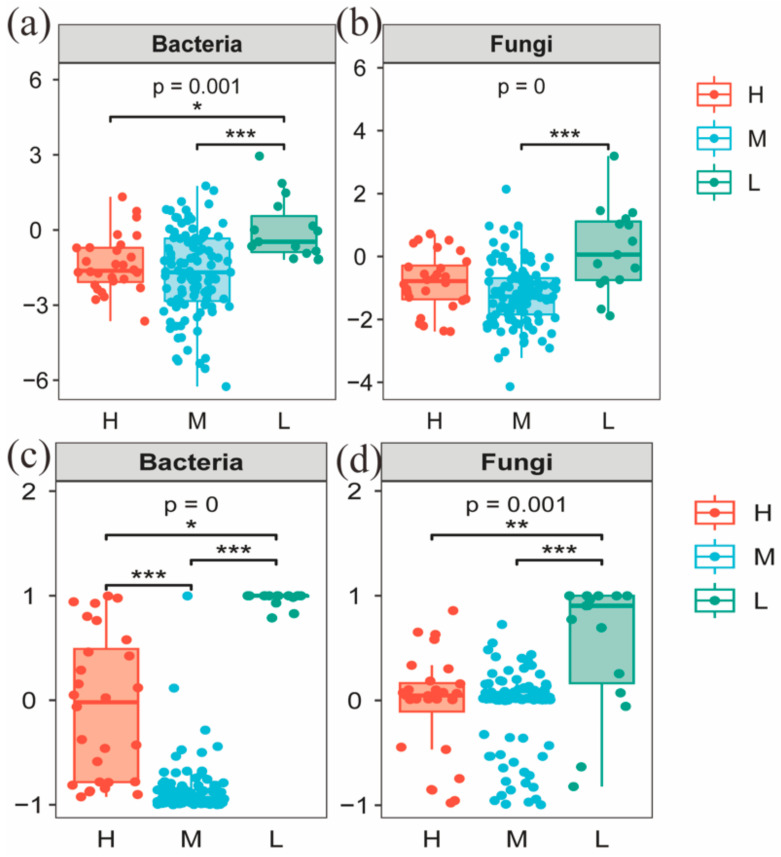
Distribution of β-NTI (**a**,**c**) and RCbray (**b**,**d**) in microbial communities on different water-salt gradients. Notes: H, M and L denote high, medium and low water-salt gradients, respectively; *, **, and *** in the figure indicate *p* < 0.05, *p* < 0.01, and *p* < 0.001, respectively.

**Figure 11:**
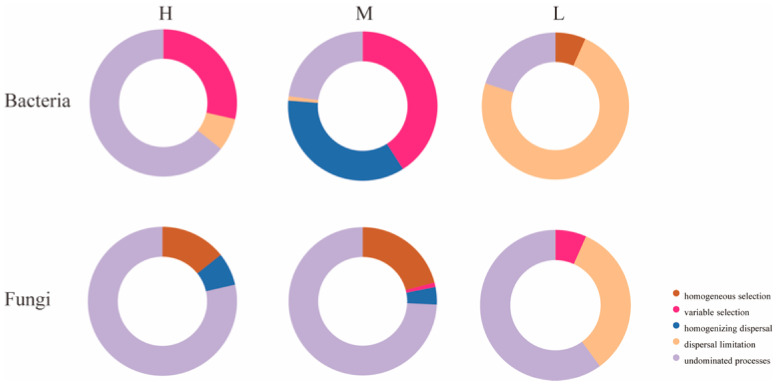
Contribution of each community assembly process under different water-salt gradients. Notes: H, M and L denote high, medium and low water-salt gradients, respectively.

To further investigate the reasons for the differences in microbial community structure under different water–salt gradients, this study used β-NTI and RCbray matrices to analyze the ecological processes of microbial community assembly. Comparisons of β-NTI and RCbray values revealed that there were significant differences in the distribution of β-NTI and RCbray values under different water–salt gradients (Figure 10). To further investigate the reasons for the differences in microbial community structure under different water–salt gradients, this study used βNTI and RCbray matrices to analyze the ecological processes of microbial community assembly. Comparison of βNTI and RCbray values revealed that there were significant differences in the distribution of β-NTI and RCbray values under different water–salt gradients (Figure 10). The undetermined processes in the stochastic process with 64.29% had a greater influence on the bacterial community under a H with 64.29%, the heterogeneous selection process with 28.57%, and dispersal limitation with 7.14% (Figure 11). Under the M, the heterogeneous selection process had the greatest influence on the bacteria with 40.95%, the homogeneous dispersal process with 35.24%, and the undetermined process with 22.86% (Figure 11). Under the L, dispersal limitation had the greatest impact on the bacterial community with 73.33% and undetermined processes with 20.00% (Figure 11).

Undetermined processes had the greatest effect on the fungal community. In addition to the undetermined processes, homogeneous selection and homogeneous dispersal had a greater impact on the fungal community in the H, accounting for 14.29% and 7.14%, respectively (Figure 11). Under the M, homogeneous selection processes accounted for 20.95% and homogeneous dispersal accounted for 3.81% (Figure 11). Under the L, dispersal limitation had a greater impact on the fungal community, accounting for 33.3% (Figure 11).

Deterministic, stochastic, homogeneous, and heterogeneous processes of microbial communities under different water–salt gradients were further analyzed (Table 4). The results show that the microbial community assembly processes were dominated by stochastic processes. In addition, the deterministic process had the greatest effect on the M and the least effect on the L. In the bacterial community assembly process, the influence of heterogeneous processes gradually increased with decreasing water–salt gradient, and homogeneous processes only existed in the M and L. In the fungal community assembly process, the homogeneity and heterogeneity processes only existed on H and M.

## 4. Discussion

### 4.1. Variation of Soil Environmental Factors along the Water–Salt Gradients

Soil water content and salinity both show a synergistic effect of gradually decreasing with increasing distance from the river bank, reflecting the basic law of water–salt transport that “salt comes with water and salt goes with water” [40]. In this study, STP, SAP, STN, SNN, SAN, SOC, and pH all decreased with the decrease in water–salt gradients, which is consistent with previous studies [41]. Soil enzymes play a key role in carbon, nitrogen, and phosphorus cycling in ecosystems, and their activities represent indicators of sensitivity to soil ecological stress or soil ecological restoration [42]. In this study, we showed that LAP, β-GC and NAG enzyme activities showed a tendency to decrease with decreasing water–salt gradients, and enzyme activities in the H were significantly higher than those in L and M, which may be due to differences in the optimum water and salt zones of different enzymes [43,44].

### 4.2. Changes in Soil Microbial Community Structure along the Water–Salt Gradients

The overall analysis of the OTU numbers showed that the bacterial OTU number (3912) were significantly higher than the fungal OTU number (1312). Among them, the number of bacterial and fungal endemic OTUs at all three water–salt gradients were in the order of L > H > M, which may be caused by the difference between environmental gradients. An increase in soil-specific microorganisms occurred with changes in water–salt content compared to the M (Figure 3a,b). The soil microbial communities differed in composition at the phylum level on the three water–salt gradients, and this study shows that there were 34 phyla of bacteria, with Actinobacteriota and Proteobacteria as the dominant phyla, and 10 phyla of fungi, with Ascomycota and Basidiomycota as the dominant phyla, in the desert soils of the Ebinur Lake basin. This is consistent with the previous studies [21,45,46]. In addition, the relative abundance of Mucoromycota exhibited significant divergence (*p* < 0.05) among water–salt gradients. The relative abundance of Actinobacteriota exhibited H < M, which may be related to the fact that most members of Actinobacteriota are less salt-tolerant and more susceptible to high salt stress [47,48]. The relative abundance at the level of bacterial genera significantly varied between water–salt gradients, with Rubrobacter, Truepera, Sphingomonas, Blastococcus, Arthrobacter and Pontibacter showing a significant increase (*p* < 0.05) with decreasing water–salt gradients. In addition, Aspergillus, Alternaria, Knufia, Penicillium and Gibberella showed significant changes with the water–salt gradients. Soil microbial community diversity exhibited a significantly higher L than H and M, which may be due to the fact that salt stress at the height of H and M reduced the microbial community diversity index [49].

### 4.3. Relationship between Soil Microbial Community Structure and Environmental Factors

Changes in environmental factors have a significant impact on the structure of soil microbial communities [50]. Soil is the most important site for microbial activity, and changes in its physicochemical properties have a significant impact on microbial community structure [51]. Soil pH is the environmental factor that plays the most significant role in microbial community structure and microbial distribution, and there is a significant correlation between the microbial community structure and soil pH [52,53,54]. In the present study, soil microbial community structure was found to be closely related to environmental factors, and pH was the environmental factor that had the greatest effect on the microbial community. SOC is also a major environmental factor affecting microbial community distribution. Previous studies found that microorganisms can obtain energy through the decomposition of organic matter and produce chemicals such as glutamine and proline in response to salt stress [55,56]. In addition, SNN, SAN, STN, and STP are also important environmental factors affecting the structure of soil microbial communities, which is in general agreement with the studies of Lauber and Kennedy [57,58]. In this study, we found that key species of soil microbial co-occurrence networks under different water–salt gradients were significantly correlated, mainly with STN and SAN, indicating that STN and SAN have a greater influence on key species, and additional experiments will be conducted in the future to enhance the study of the mechanisms of key species regulation by environmental factors.

### 4.4. Characteristics of Soil Microbial Co-Occurrence Networks under Different Water–Salt Gradients

The co-occurrence network of soil microbial communities drives the development of co-occurrence and diversity among different organisms, reflecting the ecological niche processes of microbial communities [59]. Between nodes, both positively and negatively correlated edges play important roles in microbial community structure formation and promoting soil nutrient cycling [60]. Previous studies have shown that positive correlations between nodes indicate more synergistic relationships in microbial communities [61], while negative correlations indicate competition in co-occurrence networks [62]. The results of the present study show that there were only positively correlated edges on the H and M for both bacterial and fungal communities, indicating that soil microbial communities prefer to coexist in a synergistic mutualistic manner on these gradients. In addition, based on the present results, it can be speculated that more synergistic relationships in microbial communities on H and M may play an important role in resistance to salinity stress, due to the fact that synergism facilitates the formation of more trophic levels for more efficient soil nutrient utilization, thus improving the capacity of microbial communities for material cycling, energy flow, and information transfer [63]. In co-occurrence networks, modules are more likely to have independent ecological niches [64,65]. The absence of distinct bacterial community network modules on H and M suggest that interactions between bacterial communities may not be stable on H and M, while in contrast, fungal communities form highly modular fungal interactions networks, which may be related to the different adaptations of bacteria and fungi to environmental factors.

In addition, the specificity of the soil microbial community across the water–salt gradients is reflected in the differences in key species. In this study, key species were identified using the betweenness centrality of network analysis, which is an important indicator to characterize network connectivity [66]. Key species are considered to be species that have a critical impact on the ecosystem disproportionate to their abundance, and the results of this study indicate that there are different key species on different water–salt gradients, further demonstrating the influence of habitat specificity on soil microbial community assembly. On the H, the three key bacterial genera are Woeseia, Haliangium, and norank_f_Balneolaceae, and the three key fungal genera are Chaetomium, Neocamarosporium, and Lophotrichus. On the M, the three key bacterial genera are Paracoccus, norank_f_Microtrichaceae, and Microvirga, and the three key fungal genera are Engyodontium, Alternaria, and Unclassified_o_pleosporales. On the L, the three bacterial key genera are unclassified_c__Bacteroidia, Methyloceanibacter, and Gemmatimonas, and the three fungal key genera are Alternaria, Sigarispora, and Chaetosphaeronema. Woeseia has a parthenogenic chemoautotrophic potential and may play an important role in the oxidative decomposition of hydrocarbons and inorganic sulfur compounds [67]. Haliangium, a species of mucilaginous bacteria has a strong capacity for organic matter decomposition [68]; therefore, Haliangium may be beneficial as a keystone species on the H for the balance of soil organic matter content. Balneolaceae can survive in highly stressed soils with a high salinity and structural stagnation [69]. Chaetomium has a significant probiotic effect on plant growth as well as biomass [70], and Li Ling et al. [71] showed that inoculation with Chaetomium significantly increased the relative water content, soluble protein content, and antioxidant enzyme activity of bitter melon seedlings. Neocamarosporiu is typically salt-tolerant and is commonly found in environments associated with saline plants [72]. Most taxa in Paracoccus are aerobic denitrifying bacteria that can survive in nitrate or in atmospheric oxygen environments [73]. Denitrifying bacterial strains without N2O production and reduction capacity, as well as N2O-reducing strains with N2O production capacity, have been isolated from the genus Microvirga [73], and it was shown that soil pH is a key factor in determining the biogeographic distribution of Microvirga [74]. The genus Engyodontium has ligninolytic activity and can degrade complex aromatic organic matter [75]. Alternaria include numerous taxa of saprophytic, endophytic and pathogenic species that cause diseases in a wide range of plants [76]. Methyloceanibacter are methylotrophic salinophilic bacteria that decompose organic matter [77]. Gemmatimonas have unique properties in nitrogen assimilation and allotrophy, and, in this study, Gemmatimonas acted as keystone species on the L and may have promoted nitrogen fixation on the L [78]. Not all of these keystone species were dominant, and some were rare species with a low abundance. This is in general agreement with previous studies [79]. In summary, it is evident that different key species are shaped under different water–salt gradients, and all of these key species can play a role in soil nutrient cycling to some extent, thus maintaining the balance of the ecosystem.

### 4.5. Mechanisms of Soil Microbial Community Assembly under Different Water–Salt Gradients

Deterministic and stochastic processes are important processes that drive the formation of soil microbial diversity and distribution patterns. Deterministic processes can enhance microbial functions in specific habitats, while stochastic processes can generate more diverse ecological functions to maintain the stability and continuity of ecosystem functions [14] and have positive feedback mechanisms for sustainable ecosystem functions [80,81]. It has been shown that changes in moisture can have a large impact on the soil microbial community [82]. Therefore, the relatively high stochastic processes in the L in this study provided richer species diversity and ecosystem functions. This same conclusion was verified by the results of microbial community diversity along the water–salt gradients. For both bacterial and fungal communities, alpha diversities were significantly higher in the L than in the H and M (Figure 4). In this study, stochastic processes dominated the microbial community assembly process at different water–salt gradients, further confirming that stochastic processes tend to occur in small-scale spaces of environmental change [83]. It has been shown that stronger biogeographic patterns of microbial communities can lead to a higher diffusion limitation, while lower diffusion rates of microbial communities can cause microbial community variability [84,85]. The percentage of diffusion-limited processes in the soil microbial community on the L in this study was significantly higher than on the H and M, and it had a higher alpha diversity index on the L, supporting the conclusion that lower diffusion rates of microbial communities can cause variation in microbial communities. In addition, it has been shown that the relative contributions of deterministic and stochastic processes in microbial community assembly vary at different geographical scales [86]; therefore, in future studies, it is important to increase the sampling volume as well as to consider the mechanisms of soil microbial community assembly in arid zones at larger scales. In summary, stochastic processes dominate the soil microbial community assembly process in the study area, and the relative importance of stochastic processes varies under different water–salt gradients.

## 5. Conclusions

Soil environmental factors as well as soil microbial community structure characteristics and assembly mechanisms in this study area significantly varied between water–salt gradients. Changes in water and salt altered the composition of soil microbial communities, and the soil microbial co-occurrence networks on different gradients had different characteristics. The contribution of each ecological process in soil microbial community assembly at different water–salt gradients was also significantly different. This highlights the close coupling between soil environmental factors and soil microbial community structural characteristics as well as assembly mechanisms on water–salt gradients. The next work will further expand the scale of sampling to investigate the structural characteristics and assembly mechanisms of soil microbial communities in deserts and other types of soils on a larger scale for better ecological conservation and restoration work.

## Figures and Tables

**Table 1:** Correlation analysis between indicators characterizing the structure of soil bacterial communities and soil chemical properties.

	OTUs	Chao	Shannon	Bray_Curtis	βMNTD
STP	0.034	−0.271	−0.224	0.057	0.062
SAP	0.323	−0.101	−0.045	−0.086	−0.151
STN	0.275	−0.025	0.091	−0.069	−0.060
SNN	0.093	−0.296	−0.098	0.087	0.005
SAN	0.091	−0.331	−0.158	−0.023	−0.090
SOC	0.375 *	0.083	0.126	−0.078	−0.126
pH	−0.158	−0.494 **	−0.383 *	0.146 *	−0.082
LAP	0.468 *	0.108	0.176 *	−0.137	−0.137
Β-GC	0.432 *	0.090	0.202	−0.086	−0.104
NAG	0.394 *	0.086	0.154	−0.101	−0.101
AKP	0.418 *	0.180	0.215	−0.109	−0.123

Note: * and ** denote *p* < 0.05 and *p* < 0.01, respectively.

**Table 2:** Correlation analysis between indicators characterizing the structure of soil fungal communities and soil chemical properties.

	OTUs	Chao	Shannon	Bray_Curtis	βMNTD
STP	−0.079	−0.332	−0.267	−0.006	0.111
SAP	−0.064	−0.224	−0.136	−0.013	0.611 *
STN	−0.094	−0.060	−0.138	−0.091	0.026
SNN	−0.383 *	−0.371	−0.317	0.083	−0.009
SAN	−0.164	−0.440	−0.390 *	0.129	0.317 **
SOC	0.138	0.005	0.005	−0.099	0.757 *
pH	−0.575 **	−0.504 **	−0.489 **	0.074	0.045
LAP	0.131	−0.048	0.018	−0.130	0.649 *
β-GC	0.256	−0.802	0.041	−0.047	0.611 *
NAG	0.238	−0.034	0.042	−0.084	0.789 *
AKP	0.255	0.050	0.104	−0.062	0.815 *

Note: * and ** denote *p* < 0.05 and *p* < 0.01, respectively.

**Table 3:** Topological characteristics parameters of microbial co-occurrence networks.

	Bacteria			Fungi		
Water-salt gradients	H	M	L	H	M	L
Nodes	287	291	292	56	61	108
Edges	14,472	21,766	6524	404	387	1043
Positive edges/%	100	100	60.8	100	100	84.0
Negative edges/%	0	0	39.2	0	0	16.0
Degree	100.85	149.60	44.69	14.429	12.690	19.310
Clustering coefficient	0.809	0.787	0.520	0.632	0.880	0.916
Modularity	0.283	0.181	1.911	0.524	0.532	0.454

**Table 4:** Deterministic, random, homogenous and heterogeneous processes of microbial community construction in different water and salt gradients.

Sample Plot	Deterministic Processes (Bacteria/Fungi)	Stochastic Process (Bacteria/Fungi)	Homogeneous Processes (Bacteria/Fungi)	Heterogeneous Processes (Bacteria/Fungi)
H	28.57%/14.29%	71.43%/85.71%	0.00%/21.43%	35.71%/0.00%
M	40.95%/21.90%	59.05%/78.10%	35.24%/24.76%	41.90%/0.95%
L	6.67%/6.67%	93.33%/93.33%	6.67%/0.00%	73.33/40.00%

## Data Availability

Not available.

## Supplementary Materials

The following supporting information can be downloaded at: https://www.mdpi.com/article/10.3390/microorganisms11041060/s1, Table S1: Methods for the determination of physical and chemical properties of soils, Table S2: Quadrats of water-salt gradients, Table S3: Analysis of the difference between soil water content and salt content along water-salt gradients, Table S4: Physico-chemical properties of soils under different water-salt gradients; Table S5. Soil enzyme activities at different water and salt gradients.
