# Supplementary material for: Structural Characteristics and Assembly Mechanisms of Soil Microbial Communities under Water–Salt Gradients in Arid Regions
Source: Microorganisms. 2023 Apr 18;11(4):1060. doi: 10.3390/microorganisms11041060 (PMC10142023; doi:10.3390/microorganisms11041060)
Supplement: Supplementary file 1 [file microorganisms-11-01060-s001.zip › microorganisms-2310674-supplementary.pdf]

Table S1. Methods for the determination of physical and chemical properties of soils

Soil factors	Methods	Instruments
pH	Glass electrode method	pH meter
Soil water content	Drying and weighing method	Ovens; 10,000 ppm balances
SOC	Potassium dichromate external heating method	Oil baths
STP	$HClO_4$-H_2SO_4-molybdenum-antimony anti-colorimetric method	Spectrophotometer
SAP	0.5 mol $NaHCO_3$ leaching molybdenum-antimony anti-colorimetric assay	Reciprocating oscillators; spectrophotometers
STN	Semi-micro Kjeldahl method	Autoclaves; semi-micro nitrogen fixing stills; burettes; thermostats
TNN	Phenol Disulfonic Acid Colorimetric Method	Reciprocating oscillator; Spectrophotometer
SAN	2 mol-L^{-1}KCl leaching-indophenol blue colorimetric method	Reciprocating oscillator; Spectrophotometer

Table S2. Quadrats of water-salt gradients

Water-salt gradients	Corresponding sample numbers
H	C1、C2、C3、C5、C7、C9、C10、C18
M	C4、C6、C8、C11、C12、C13、C14、 C15、C16、C19、C20、C21、C22、C24、C25
L	C23、C26、C27、C28、C29、C30

Table S3. Analysis of the difference between soil water content and salt content along water-salt gradients.

	Soil water content（%）	Maximum value（%）	Minimum value（%）	Soil salinity（g·kg^{-1}）	Maximum value（g·kg^{-1}）	Minimum value（g·kg^{-1}）
H	14.353±1.251a	19.664	10.680	8.289±1.002a	12.201	6.290
M	7.797±1.022b	12.431	5.663	3.526±0.124b	4.613	2.851
L	3.193±0.254c	5.229	1.822	1.596±0.056c	2.304	0.481

Table S4. Physico-chemical properties of soils under different water-salt gradients, Note: Different letters in the table for the same physicochemical factor indicate significant differences between groups (p<0.05)

	SOC (g/Kg)	STN (mg/Kg)	SAN (mg/Kg)	SNN (mg/Kg)	STP (mg/Kg)	SAP (mg/Kg)	pH
H	18.80±4.38a	587.81±71.38a	3.43±0.33a	3.10±0.08a	405.12±47.79a	60.01±9.44a	8.81±0.05a
M	9.04±0.47a	228.14±28.77b	2.36±0.20b	2.87±0.15a	271.57±8.11b	26.17±3.82b	8.66±0.02b
L	7.12±1.27b	240.06±38.83b	1.40±0.16c	2.13±0.24b	203.70±8.36b	9.12±1.68b	8.45±0.05c

Table S5. Soil enzyme activities at different water and salt gradients, Note: Different letters in the table for the same physicochemical factor indicate significant differences between groups ($p < 0.05$)

	LAP (μmol/d/g)	β-GC (μmol/d/g)	S-NAG (μmol/d/g)	AKP (μmol/d/g)
H	15.16±3.01a	13.65±3.20a	18.28±4.16a	4.16±1.27a
M	6.33±0.37b	6.61±1.17b	10.19±0.36b	1.97±0.04b
L	5.34±0.81b	4.30±2.21b	8.67±0.31b	1.97±0.38b
